# Haemoglobin revisited: delineating population structure with the world’s first molecular genetic marker used in fisheries research

**DOI:** 10.1098/rsos.241760

**Published:** 2025-01-15

**Authors:** Bjørghild Breistein, Geir Dahle, Torild Johansen, Per Erik Jorde, Kevin A. Glover

**Affiliations:** ^1^ Institute of Marine Research (IMR), PO Box 1870, Bergen N-5817, Norway

**Keywords:** *Gadus Morhua*, Atlantic cod, haemoglobins, population genetics

## Abstract

When haemoglobin genotyping was implemented in the early 1960s to investigate population genetic structure in Atlantic cod (*Gadus morhua*), it became one of the first molecular genetic markers deployed in fisheries research worldwide. However, its suitability was questioned due to its potential for selection. While the issue of neutrality concerned the first population geneticists, markers under selection are now routinely used to study population genetic structure. Here, we revisited haemoglobin genotyping half a decade later to analyse >6000 mature Atlantic cod from 73 spawning locations throughout Norway’s approximately 2500 km coastline. A latitudinal gradient in allele frequencies, with a decrease in the HbI-2 allele towards the south, was observed. Our observed HbI-2 frequencies were consistently slightly lower than data from the 1960s, potentially reflecting adaptive changes to increasing sea temperatures. However, despite this difference, the observed north–south pattern in allele frequencies observed here and in the historical studies overlapped, aligning with current knowledge of population genetic structure in this species. We therefore conclude that this once questioned marker, which provided the first molecular genetic insights into genetic structure in Atlantic cod, provides knowledge consistent with the isolation by distance pattern revealed through decades of research in this species in this region.

## 1. Introduction

Delineating population genetic structure plays an important role in the management of the world’s living resources. This is especially the case for marine fisheries resources that have been heavily targeted for harvest, leaving a legacy of extensive exploitation and overexploitation.

Atlantic cod (*Gadus morhua*) is a commercially important demersal fish that has formed the basis of historically major fisheries throughout the North Atlantic, some of which are heavily depleted and/or collapsed [1,2]. In Norway, which is home to some of the largest remaining cod fisheries throughout its distribution range [3], the species displays two genetically and behaviourally distinct ecotypes [4–6] that are also recognized and separately managed by the regulatory authorities in this region. These are the migratory Northeast Arctic cod (NEAC) that travel long distances from their feeding grounds in the Barents Sea to the Norwegian coast during the annual spawning season and the local and non-migratory Norwegian Coastal cod (CC) that reside in the fjords along the coast.

Historically, Rollefsen [6] identified structural otolith differences between what we now recognize as the NEAC and CC ecotypes, a method that has subsequently been used as the primary tool to discriminate between them in stock assessments [7]. In the early 1960s, the world’s first genetic marker to delineate a major fishery resource was developed. This was achieved by Sick [8] who showed that Atlantic cod haemoglobins could be separated by agar gel electrophoresis into two main components, HbI and HbII, the first component of which primarily showed two alleles HbI-1 and HbI-2 that were detectable using this technology [8]. Shortly after this discovery, the geographical distribution of the HbI alleles in samples of cod on the eastern side of the North Atlantic was investigated, revealing a distinct clinal reduction in the HbI-1 allele frequency with increasing latitude from 69.0% to 7.3%, although a much less clear north–south gradient was found on the western side [9,10]. Additionally, allele frequency differences were detected between CC and NEAC in Norway [11]. In the 1990s, the haemoglobin analysis of Atlantic cod from the Barents Sea and some spawning sites along the northern Norwegian coast showed both a close correspondence with results from the 1960s and a strong correlation between the otolith type and the HbI-1 allele [12]. The HbI-1 allele frequency along the Norwegian coast has more recently been reported [13], revealing a gradient ranging from 7.5% in the Barents Sea to 62.5% in the southern population at Helgoland, supporting earlier findings.

There has been an increase in knowledge of haemoglobin complexes since their initial discovery. The three tetramers in adult cod (HbI, HbI and HbIII) were purified and found to comprise different combinations of the four subunits α1, α2, β1 and β2 [14]. Additional globin genes are found in the Atlantic cod genome, where they are located in two unlinked clusters β5-α1-β1-α4 and β3-β4-α2-α3-β2, on chromosomes 2 and 18, respectively. These subunits are expressed at different times during the development stages, but more research is needed to fully understand these patterns [15–17]. The most studied tetramer, also examined in this study, HbI, consists of the four subunits α1, α1, β1, β1, that are genetically located in the chromosome 2 cluster. The root of the difference between the two main HbI versions, HbI-1 and HbI-2, is two amino acid substitutions located in the β1 subunit, which causes functional differences in the protein structure, and thus a different affinity for oxygen. The subunit found in the HbI-2 type has a lower affinity for oxygen in higher temperatures but is the most efficient at lower temperatures [13,15,18]. In addition, the protein is also affected by temperature through a secondary mechanism, namely the expression of it. The joint promoter region that controls the expression is located between the genes coding for the α1 and the β1 subunits and exists in two versions, the long and short variants, strongly correlated to the HbI-1 and HbI-2 variants [16,19]. This promoter has shown in experiments that the long version connected to HbI-2, which is more abundant in the north, increases the expression of the protein twofold in response to temperature increase, compared with the short version, thus potentially compensating for the lower oxygen affinity [19].

Despite the historically significant first insights into population genetic structure revealed by haemoglobin analysis [8], its suitability as a genetic marker to quantify population genetic structure was later questioned when it was revealed that the different alleles had different oxygen-binding properties at different temperatures [20–22]. The potential for selection to influence haemoglobin allele frequencies therefore led some [23] to stipulate that the observed north to south genetic gradient in allele frequencies may be at least in part influenced by selection rather than reflecting a purely demographic pattern of population structure and connectivity. However, while the question of neutrality was of great concern to the early population geneticists [24,25], many marine resources are now routinely split and identified as populations using markers under sometimes strong selection and adaptive significance [26,27]. This is because such markers are often the only ones permitting delineation between large marine populations where the process of genetic drift often fails to leave an imprint on the genome, especially in the face of high levels of gene flow.

In the present study, we revisited the world’s first molecular genetic marker used in fisheries research to create a detailed map of allele frequency in CC along Norway’s entire coastline. The main objectives of this work were to first, investigate the population genetic structure of CC in Norway’s coastline given by HbI allele frequencies, and relate this directly to the historical observations made approximately 40−50 years earlier by using the same genotyping technology, and second, to compare the results against our current understanding of population genetic and genomic structure of Atlantic cod in this region [3,28–30]. These objectives were achieved by analysing HbI in 6140 adult Atlantic cod that were collected from 73 spawning locations spanning approximately 2500 km of Norway’s coastline in the period 2002−2007 by using traditional agar gel electrophoresis.

## 2. Material and methods

**Figure 1.**
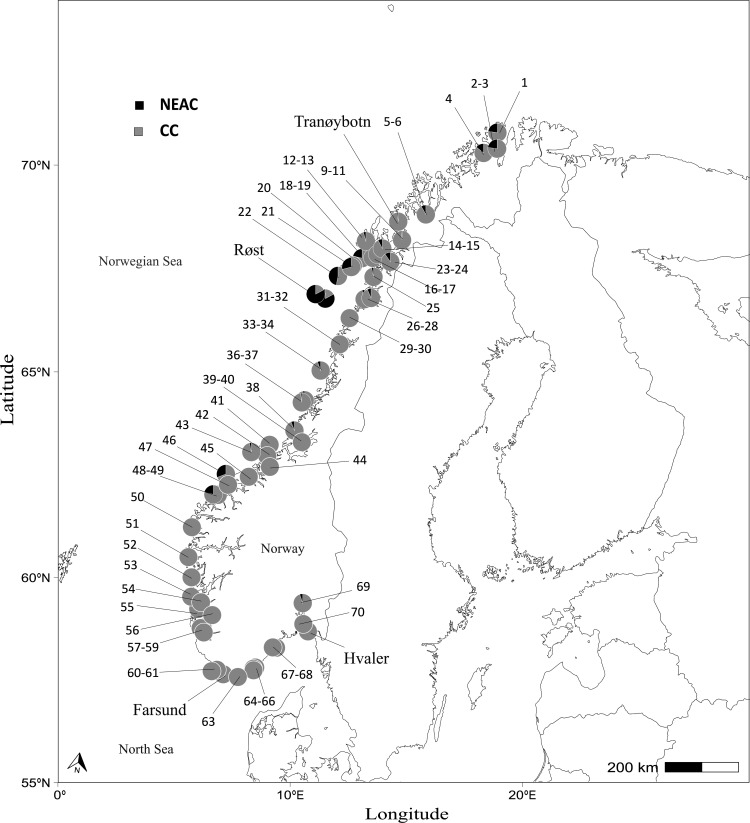
Sampling locations for Atlantic cod along the Norwegian coast. Numbers on the map correspond to the numbering of stations in electronic supplementary material, table 1. Colour (black and grey) in the pies illustrates the proportion of NEAC in each sample, based upon scoring otoliths. Locations over multiple years are compiled. The following four samples are mentioned in the manuscript text: Tranøybotn (7-8), Røst (72-73), Farsund (62) and Hvaler (71).

In the spawning seasons of 2002−2007, a total of 6140 running or spent Atlantic cod were sampled at known spawning sites along the Norwegian coast (figure 1, electronic supplementary material, table S1). From each individual, approximately 1 ml of blood was collected, in addition to the following: otoliths, length, sex and maturity status (spawning or spent). Blood samples were placed into separate wells in a 96-well Nunc ™ tray that contained heparin to prevent coagulation. These samples were thereafter analysed within 24 h using agar gel electrophoresis using a protocol described by Jørstad [8,31]. Typically, two haemoglobin components can be distinguished using this method. The strongest zone is produced by the products of the HbI locus and its two main alleles: HbI-1 and Hb1-2. In addition, a third very slow-moving allele was observed at a very low frequency, earlier reported in several studies [9,12].

Otoliths were collected from the genotyped individuals and scored by experienced readers into one of five categories: (i) certain CC, (ii) uncertain CC, (iii) Svalbard cod, (iv) uncertain NEAC and (v) certain NEAC. Individuals were thereafter grouped as follows: (i) and (ii) CC and (iv) and (v) NEAC [7]. Less than 0.1% of the 6140 individuals (five individuals) were characterized as type 3 and were therefore omitted from all subsequent analyses along with individuals where type could not be determined (thus, only individuals categorized as 1, 2, 4 or 5 were included). For the analyses of genetic structure, individuals without haemoglobin scores were omitted, in addition to two stations that primarily consisted of NEAC as revealed by otolith scoring. The proportion of NEAC in samples, as revealed by the otolith category, was visualized spatially as pies on a map through the R package *mapmixture* [32].

**Figure 2.**
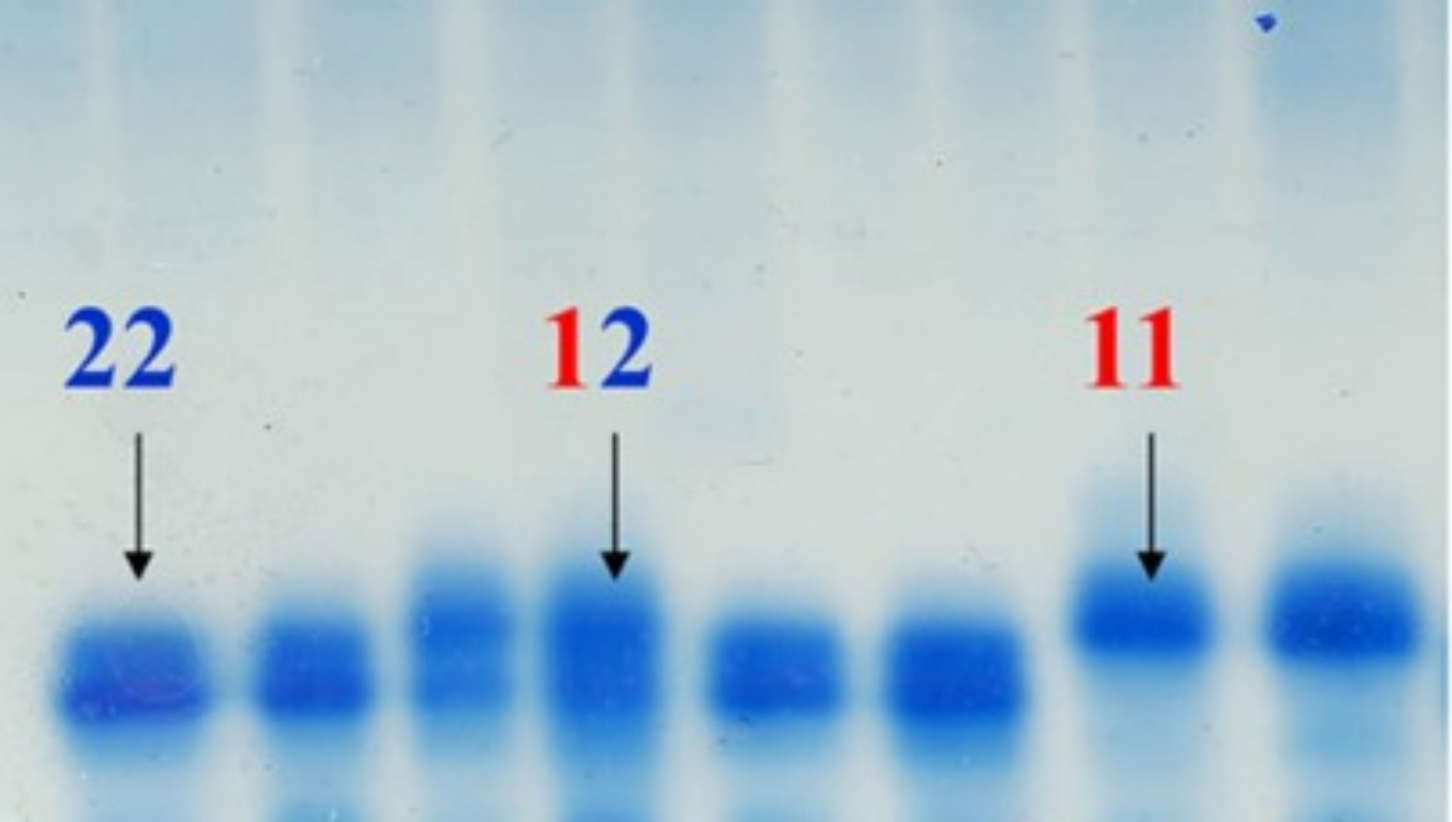
Photograph of an agar gel used to score haemoglobin in both the 1960s and the present study. HbI alleles are labelled as the 1/1, 1/2 and 2/2 genotypes.

HbI allele frequencies were computed per population from all individuals captured at that location, and in addition, for all individuals captured at that location and categorized as CC by otolith reading (thus removing NEAC). An example of the allele scoring is presented (figure 2). Allele frequency was calculated in the R package *adegenet* [33]*,* and deviations from Hardy–Weinberg genotype proportions were characterized by *F*_IS_ and calculated within the R package *hierfstat* [34].

The R package *marmap* [35–37] was used to calculate the closest sea-distance matrix among all sampling locations, which was thereafter used to test for genetic isolation by distance (IBD) through a Mantel test in the R package *ade4* [38,39]. Pairwise estimates of genetic divergence, *F*_ST_ for the IBD test were computed with *PopgenReport* [40].

Allele frequencies resulting from the present study were compared to historical data from Frydenberg *et al*. [9]. The Frydenberg study had no otolith-based removal of NEAC individuals. Therefore, to make our data directly comparable to the historical data, NEAC were also kept in our data for this specific comparison. In addition, the two stations dominated by NEAC in the present study (Røst and east of Røst) were included in the comparison to the historical data. The allele frequency of HbI-1 relative to the distance to Hvaler was used to compare the two studies. Locations where more than one independent sample was taken were combined, and for stations without coordinates in the Frydenberg study, the description of sampling sites was used to determine the approximate location. The R package *marmap* was used to find the closest sea-distance, and the results were visualized in the R package *ggplot2* [41]. The relationship between HbI-1 allele frequency and geographic distance was tested by comparing several linear models, lm() in R. All models included HbI-1 allele frequency as a response variable to geographic distance and dataset (dataset1 includes samples from the year 1961 to 1963 and dataset2 sampling year from 2002 to 2007). We considered the possible effect of the datasets (sampling period) as well as an interaction between the dataset and geographic distance, leading to a different regression slope between the two sets. Models were compared with ANOVA() and model parameters were kept based on the likelihood ratio test with a significance threshold of *p* = 0.05.

To spatially visualize the historical and contemporary datasets, allele frequencies were plotted on separate maps in *mapmixture*. The sample representing NEAC for the contemporary data (i.e. our study) included all the NEAC individuals identified by otoliths from the different locations.

## 3. Results and discussion

The proportion of individual NEAC in the 73 samples taken along the Norwegian coastline, based on the otolith category of 5669 cod, is presented (figure 1). It shows that NEAC were present in parts of Northern Norway, especially the Lofoten Islands, and are all but non-existent in the south of Norway. Although five individuals in Oslofjorden were categorized as uncertain NEAC, these individuals are most likely misread rather than true NEAC. The observed patterns in the distribution of NEAC thus overlap with the reported spawning grounds for NEAC, and the fact that below 62° N, NEAC are absent [30].

The final haemoglobin dataset consisted of 5273 and 294 adult CC and NEAC, respectively (identified by otolith classification), collected from 71 spawning sites covering approximately 2500 km of the Norwegian coastline (this excludes the samples from Røst and east of Røst that were predominantly NEAC; see figure 1). For the analysis including only otolith identified CC, the frequency of the HbI-1 allele ranged from 0.23 (in Tranøybotn, in the north; figure 1 and electronic supplementary material, table S1) to 0.73 (in Farsund, in the south; figure 1 and electronic supplementary material, table S1). The inclusion of NEAC in the CC samples did not change these observations notably (see electronic supplementary material, table S1).

**Figure 3.**
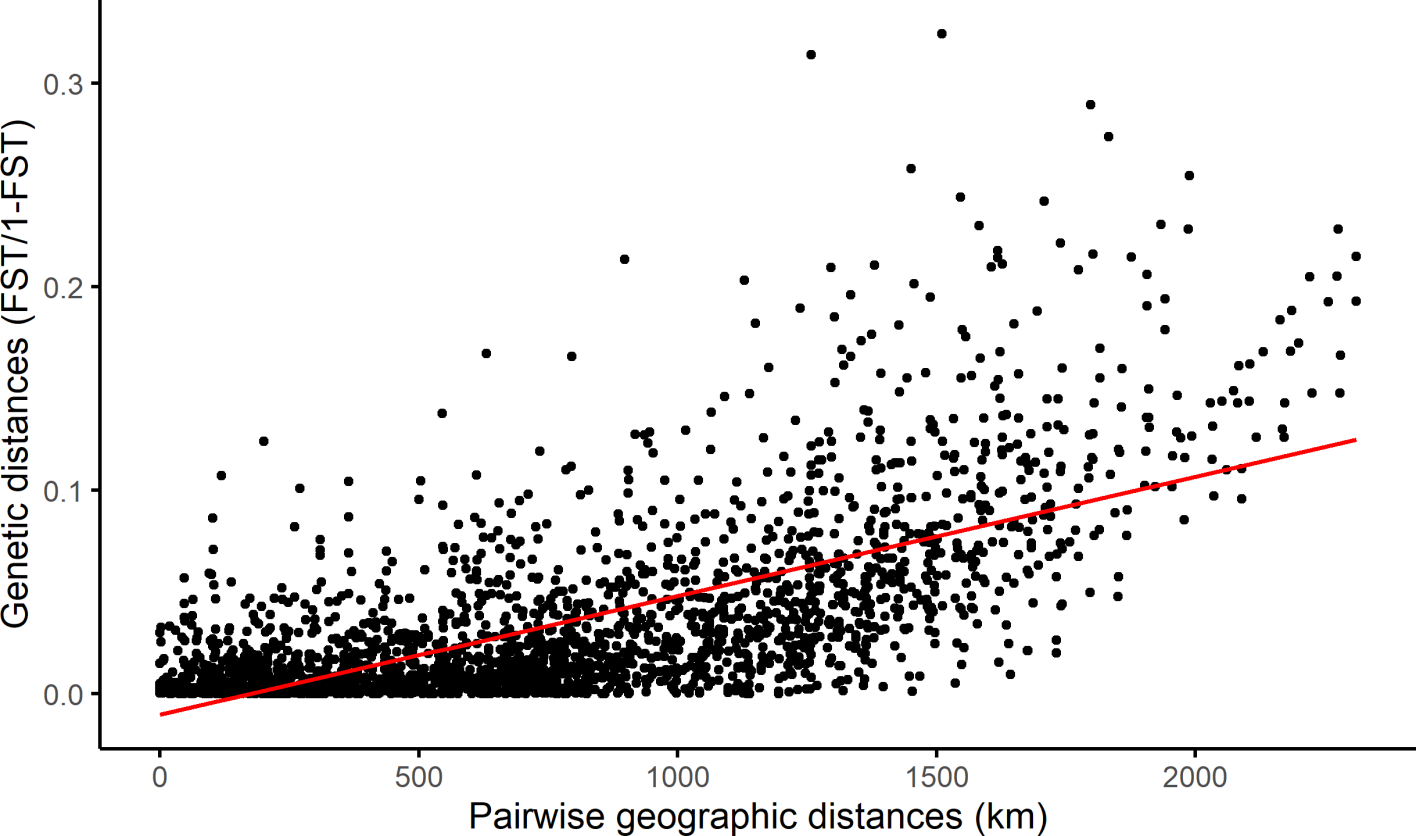
Isolation-by-distance plot of genetic distance for coastal cod (NEAC not included) based on haemoglobin frequencies, plotted against the closest sea-distance, mantel estimated *r* is 0.68 with a *p*-value of 0.001.

**Figure 4.**
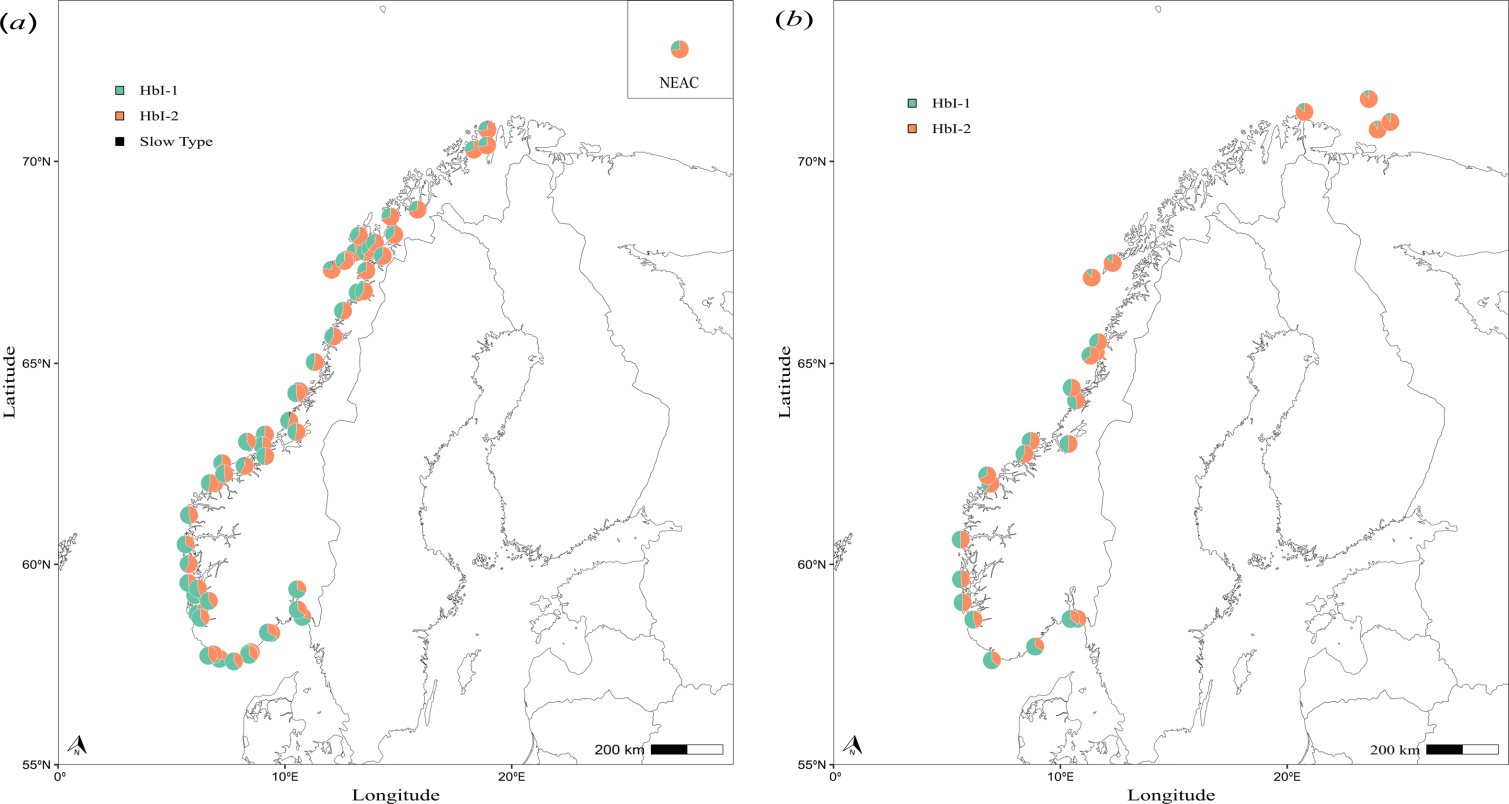
Frequencies of the HbI-1 and HbI-2 alleles in cod from the present study (*a*) and extracted from a study in the early 1960s [9] (*b*). Both datasets contain coastal and NEAC individuals. Sites in very close geographical proximity and/or sites sampled over multiple years are aggregated. The pie illustrating the allele frequencies for otolith-identified NEAC in the present dataset (depicted by the square inset upper-right in panel (*a*)) is a compilation of the individual fish identified as NEAC in all locations. For samples lacking exact coordinates in Frydenberg *et al*. [9] (*b*), the description of the sampling location was used to decide placement on the map and is thus approximate.

Allele frequency data displayed a strong pattern of genetic isolation by distance, in the form of a cline from north to south (figure 3). The cline was almost identical whether computed on the material consisting of all fish or only the NEAC otolith-purged material. The gradient shown is highly consistent with our current understanding of the population genetic structure of CC in Norway, that is, a north to south gradient of genetic connectivity, based on the analysis of microsatellites [42] and genome-wide SNPs [28–30]. Collectively, these studies illustrate that the cline in population genetic structure within CC is at least in part driven by interbreeding with the genetically distinct NEAC that occurs most in the north and less in the south. This is illustrated by the fact that CC are genetically more similar to NEAC in the north (figure 4), and is especially noticeable in the Pan I gene [43,44] that is located in the large genomic inversion on chromosome 1 [4]. However, it is also likely that at least part of this cline may also reflect adaptation to the north–south temperature gradient [21]. In support of this possibility is the fact that cod in the Baltic Sea, which are further south than the southernmost location in this study but experience far colder temperatures, are dominated by the HbI-2 allele [13]. Clearly, further genomics work is needed to disentangle the relative influence of these two forces.

The genes coding for HbI are located approximately 3 Mb upstream of the reported inversion on chromosome 2 that displays a genetic gradient on both sides of the Atlantic and has, among other things, been linked with potential temperature adaptations [45–47]. Haemoglobin is potentially also under temperature selection in Atlantic cod due to the differences between the alleles in their temperature-dependent oxygen-binding affinities [13,20,21], with links to temperature preference [22,48] and growth [21]. Whether the adaptation profile for the inversion on chromosome 2, and haemoglobin itself located outside of this inversion, coincide or is conflicting poses an interesting question. However, further research is needed to identify whether other genes in the inversion, e.g. the genes associated with adaptation to salinity [49,50], would drive adaptation in a different direction than haemoglobin. If this were the case, it may provide an explanation as to why haemoglobin has not been included in the inversion itself.

The greater oxygen-binding affinity at lower temperatures for the HbI-2 allele observed within the studies mentioned above is consistent with both the historical [9,10,12] and the present study’s observations in geographic patterns for its allelic distribution, i.e. the observed increase in HbI-2 in the north, underpinning a strong genetic cline. Based upon his initial work, Sick [10] concluded that different selective forces in different areas, i.e. recent and current environmental conditions, in combination with the distributional history of the species, resulted in the heterogeneous distribution he observed. It would thus appear that his interpretation of the first molecular genetic fisheries data was surprisingly accurate and certainly consistent with current knowledge. Thus, despite potentially strong selection and changing environmental conditions across nearly five decades, markers such as haemoglobin can give reliable pictures of population genetic structure.

**Figure 5.**
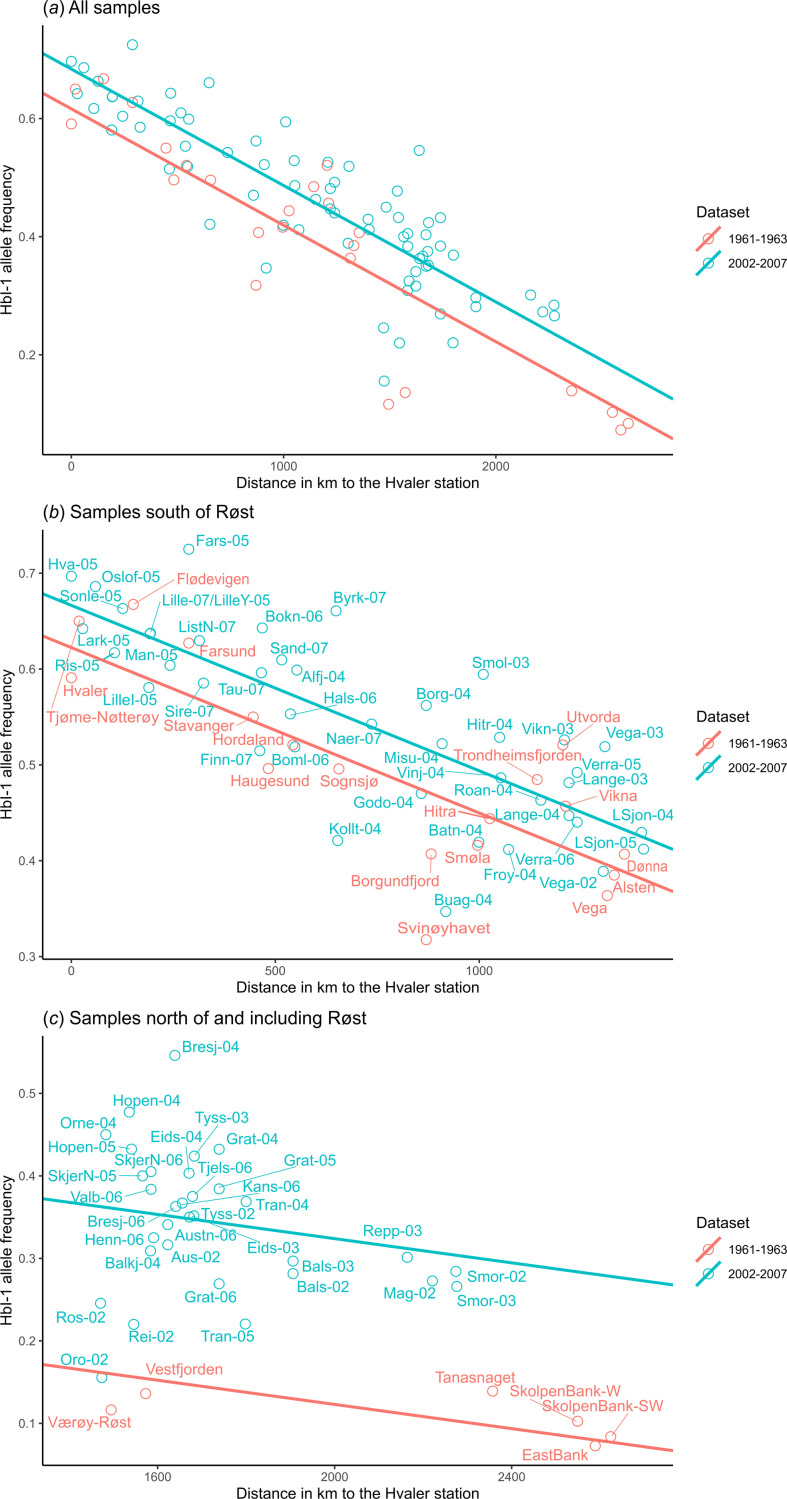
Frequency of the HbI-1 allele relative to the distance in kilometres to the station in Hvaler, for the historical data from Frydenberg *et al*. [9] (1961–1963) and for the contemporary data in the present study (2002–2007). Panel (*a*) covers all samples while panels (*b*) and (*c*) represent a zoom-in on geographic regions. The regression model (HbI-1 ~ distance + dataset) explains a significant portion of the variance for all samples combined (*a*: *R*² = 0.77, *p* < 0.001), for all samples south of Røst (*b*: *R*² = 0.77, *p* < 0.001) and all samples including and north of Røst (*c*: *R*² = 0.59, *p* < 0.001).

As mentioned, we observed a consistent trend of decreasing HbI-1 allele frequency with geographic distance in both historical and contemporary samples (d.f. = 1; *F* = 268.71; *p* < 0.001); however, the interaction between dataset and geographic distance was not significant (d.f. = 1; *F* = 2.31; *p* = 0.13). This means that the north–south trend was not significantly different between the 1961−1963 samples and the 2002−2007 samples. The dataset effect itself was, however, significant (d.f. = 1, *F* = 15.71, *p* < 0.001), with the overall frequency of HbI-1 being lower in the 1961−1963 samples than in the 2002−2007 samples. These results are illustrated in figure 5 where the predicted model (HbI-1 ~ distance + dataset) is represented as solid lines (figure 5*a–c*). The results are statistically significant and consistent also when you split the coast into parts with more (figure 5*c*) and less (figure 5*b*) potential for NEAC in samples. The fact that the frequencies of the allele variant adapted to colder environments, i.e. HbI-2 [20–22] is generally lower in the contemporary samples in the present study represents an interesting observation. Since the 1960s, there has been a global increase in sea temperatures [51]. On the coast of Norway, sea temperatures have increased 0.03–0.04°C on average each year from 1968 to 2005, measured at 50 m depth in the last quarter of the year at Ingøy in the north (approx. 71.11° N 25.05° E), and Utsira in the south (approx. 59.31° N 4.74° E). These values were collected from the hydrographical data from the Institute of Marine Research’s fixed coastal stations (https://www.imr.no/forskning/forskningsdata/stasjoner/index.html) [52]. The observed difference in allele frequencies between the historical and contemporary samples could therefore reflect an adaptive response to the temperature change in this time period. Temperature responses have been observed in cod, both directly in behaviour analysis that showed changes in depth use [53], experimental studies where temperature increases lead to altered gene-expression [54], and also modelled how an increase in temperature reinforced management actions resulting in an all-time high NEAC stock in 2013 [55]. Arguments can be made that a decrease in the HbI-2 version, the one adapted for colder environments, is an expected outcome of the temperature increase. However, it is important to note that the HbI-2 allele has a built-in compensator for temperature increase that may buffer adaptive changes [19]. Consistent with this are the results of an experimental study that concluded that physiological plasticity was more relevant for juveniles than adaptation, by testing individual growth rate and stress level in response to temperature according to their HbI type [56].

Although temperature adaptation could have caused the temporal change in HbI allele frequencies, it is important to note that two other factors may have influenced this result. First, our biological samples were gathered during the spawning season, while the Frydenberg *et al*. [9] samples were not consistently gathered during the spawning season. This can lead to a different proportion of NEAC in some of the samples (especially in the north) and thus influence allele frequencies. In fact, the low frequencies of the HbI-1 allele, and the low variation between the stations in the north for the older samples (figure 5*c*) seem to indicate a very high proportion of NEAC in the Frydenberg samples. However, we see a consistently lower frequency in the historical samples when only southern samples with a lower potential for NEAC presence are included, suggesting that there has been a real shift in allele frequencies in relation to changing oceanic temperatures. Second, while we used traditional equipment to score haemoglobin that is comparable to the study by Frydenberg and Sick [8–10], it is impossible to exclude the possibility of systematic scoring bias between the two studies, as gels are scored manually (figure 2). While it is not possible to validate or exclude any of these explanations for the observed differences between the historical and contemporary datasets here, the fact that haemoglobin displays obvious temperature adaptive potential, and sits outside a temperature-associated inversion [46,47], certainly suggests adaptation is a possibility.

As discussed in detail in the introduction, haemoglobin is one of the best studied proteins in nature [57], showing complex thermal adaptations in its gene-cluster [19]. Intraspecific variations in haemoglobin patterns have been described in other fish species; for example, Italian catfish (*Ictalurus* spp.) [58], turbot (*Scophthalmus maximus*) [59], Nile tilapia (*Oreochromis niloticus*) [60], beaked redfish (*Sebastes mentella)* [61] and tusk (*Brosme brosme*) [62]. Although the potential for selection in haemoglobin in other marine species is present, as for Atlantic cod, analysis of this marker either alone or in association with other molecular markers can provide population genetic inferences. As initially demonstrated in [15], more Hb genes and alleles are present and expressed in adult Atlantic cod than previously documented by using protein gel electrophoresis or protein sequencing. These findings suggest that further analysis of haemoglobin polymorphisms, using more advanced analyses such as transcriptome sequencing, could provide further insights into the geographic distribution of alleles for this marker and help understand its adaptive landscape.

Evolution in the choice of molecular markers implemented in population genetic studies has followed a similar path across species and systems. Broadly speaking, it developed from proteins to mtDNA and microsatellites, and then to SNPs with a brief application of non-specific methods such as random fragment polymorphisms and similar techniques [63]. At each developmental step, technical advantages were promoted, providing researchers with the hope of revealing the previously unidentifiable genetic structure and/or unveiling novel evolutionary mechanisms of structure and adaptation. While this has often been the case, especially in the post-genomic era where structural chromosome rearrangements [5,64,65] or genes of major influence have been identified [66–68], surprisingly, many studies using the early markers still gave the ‘correct’ indication of what was later unveiled and/or validated with more advanced techniques. In addition to the work on cod here, examples exist for knowledge in Atlantic salmon (*Salmo salar*) population structure across the North Atlantic using allozymes [69], microsatellites [70] and genome-wide SNPs [71], and for example, for the European sprat (*Sprattus sprattus*) first with microsatellites [72,73], then with SNPs [74] and finally using SNPs across the whole genome [75].

## 4. Conclusions

To our knowledge, this work represents the most extensive analysis of the population genetic structure in a marine organism using haemoglobin. We show that although haemoglobin is likely to be under selection, it provides a picture of population genetics for coastal cod in Norway that is both consistent with historical results with this technique some 40−50 years earlier, and importantly, highly consistent with our current understanding of the population genetic structure in this species as also inferred from genomic studies. The finding of a modest shift in allele frequencies over this time scale is consistent with a response to rising sea temperatures but needs further research to clarify cause and effect. Overall, although results using haemoglobin were once questioned due to the issue of neutrality, the first ever interpretations of population genetic structure in a marine organism using a molecular genetic marker, by Sick in the very early 1960s, were surprisingly consistent with findings with modern techniques. This highlights the enduring value of certain historical tools and data that may still provide important insights in the modern context.

## Data Availability

The data that support the findings of this study are included as electronic supplementary material [76]. It consists of an excel file with otolith and haemoglobin scoring for 5887 individuals, an excel file containing station numbers and allele frequencies and a text file with the R code used to generate figures.
